# A case with unilateral hypoglossal nerve injury in branchial cyst surgery

**DOI:** 10.1186/1749-7221-7-2

**Published:** 2012-02-01

**Authors:** Sudipta Kumer Mukherjee, Chandra Bidhan Gowshami, Abdus Salam, Ruhul Kuddus, Mohshin Ali Farazi, Jahid Baksh

**Affiliations:** 1 Dept. of Neurosurgery-Shaheed Sheikh Abu Naser Hospital (SSANSH)-Khulna-Bangladesh; 2 Dept. of Cardiology-Shaheed Sheikh Abu Naser Hospital (SSANSH)-Khulna-Bangladesh; 3 Dept. of Neurology-Shaheed Sheikh Abu Naser Hospital (SSANSH)-Khulna-Bangladesh; 4 Dept. of Paediatric Surgery- Khulna Medical College Hospital (KMCH) - Bangladesh

**Keywords:** Hemiatrophy, Hypoglossal nerve, Brancial cyst

## Abstract

An 11 years old boy came, with complain of mild dysarthria. Examination revealed marked hemiatrophy of left side of the tongue. Five months back he underwent ipsilateral branchial cyst operation. To our knowledge, no case was reported. After branchial cyst operation if there is any residual remnant chance of recurrence is very high.

## Introduction

Injury of the hypoglossal nerve is a recognized complication following soft tissue surgery in the upper and anterior part of the neck [1,2], and [3]. Among 100 cases of hypoglossal nerve palsy (33 bilateral) reported by Keane [4], five cases were the result of a surgical procedure: for clival tumors in 2 cases, and carotid endarterectomy and a tonsillectomy in 1 case each, one case was not specified.

In this series in most patients, 12th-nerve involvement was asymptomatic or a minor component of dysarthria. On the contrary acute 12th-nerve transection causes early and late serious oral problems in children [5].

Branchial cleft (branchiogenic) cysts are congenital epithelial cysts, which arise on the lateral part of the neck from failure of obliteration of the second branchial cleft [6]. Their location makes them prone for 12th-nerve lesions, but literature mentions are scarce.

## Case report

An 11 years' old boy was operated because of a branchial cyst. The cyst was not infected but the surgeon faced the problem of tissue retraction during operation. One week after operation deviation of the tongue was noted and examination revealed marked atrophy of the ipsilateral side of the tongue. Electromyography revealed partial 12th-nerve injury. Explorative surgery of the hypoglossal nerve was regarded not to be indicated. Improvement was not noted six months after operation.

## Conclusion

Hypoglossal nerve palsy may be caused by tumors and cysts in the neck, but can also be caused by surgery. It is important to note securely the preoperative situation.

## Consent

Written informed consent was obtained from the patient for publication of this case report and accompanying images. A copy of the written consent is available for review by the Editor-in-Chief of this journal.

## Competing interests

The authors declare that they have no competing interests.

## Authors' contributions

SKM is the chief author who deals the patient clinically, BCG draws SKM attention for this case, AS & RK perform EMG, MAF help SKM in every aspect and JB take care of this patient preoperatively. All authors have read and approved the final manuscript.

## Authors information

1. Sudipta Kumer Mukherjee- Junior consultant-MS(neurosurgery).- Dept of Neurosurgery-Shaheed Sheikh Abu Naser Hospital (SSANSH)-Khulna-Bangladesh.

2. BC Gowshami--Assistant Professor - MD(Cardiology)- Dept of Cardiology-Shaheed Sheikh Abu Naser Hospital (SSANSH)-Khulna-Bangladesh.

3. Abdus salam- Junior consultant -MD (neurology)-Dept of Neurology-Shaheed Sheikh Abu Naser Hospital (SSANSH)-Khulna-Bangladesh.

4. Ruhul kuddus -Assistant Professor-MD(Neurology). Dept of Neurology-Shaheed Sheikh Abu Naser Hospital (SSANSH)-Khulna-Banglaesh.

5. Mohshin Ali Farazi -MS(neurosurgery). Registrar Dept of Neurosurgery-Shaheed Sheikh Abu Naser Hospital (SSANSH)-Khulna-Banglaesh.

6. Jahid Baksh -Assistant professor -MS(Paediatric surgery)-Dept of Paediatric Surgery- Khulna Medical college Hospital - Bangladesh.

**Figure 1:**
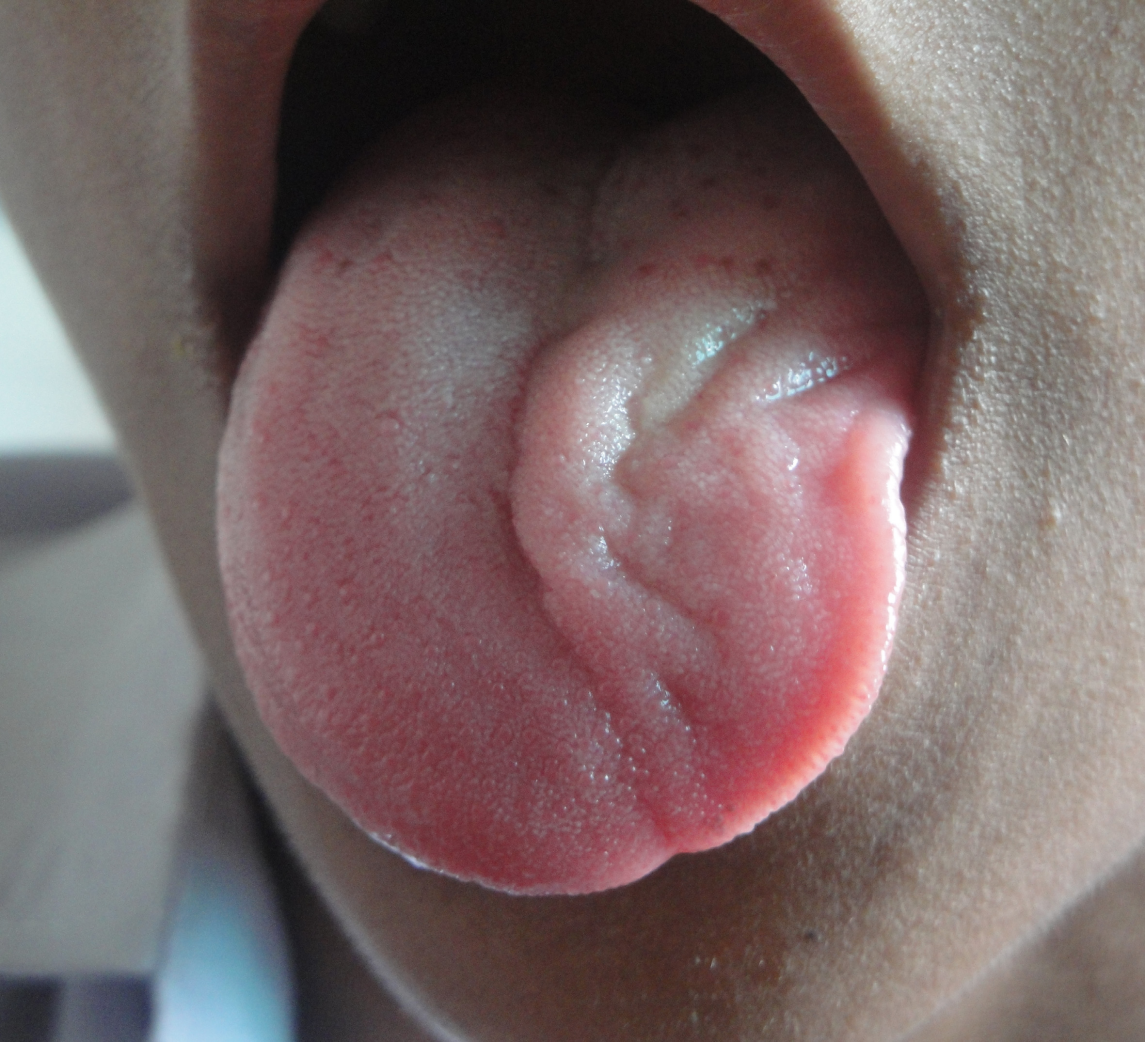
**Show marked hemiatrophy of tongue after injury of hypoglossal nerve**.
